# Development of depression detection algorithm using text scripts of routine psychiatric interview

**DOI:** 10.3389/fpsyt.2023.1256571

**Published:** 2024-01-04

**Authors:** Jihoon Oh, Taekgyu Lee, Eun Su Chung, Hyonsoo Kim, Kyongchul Cho, Hyunkyu Kim, Jihye Choi, Hyeon-Hee Sim, Jongseo Lee, In Young Choi, Dai-Jin Kim

**Affiliations:** ^1^Department of Psychiatry, College of Medicine, Seoul St. Mary’s Hospital, The Catholic University of Korea, Seoul, Republic of Korea; ^2^College of Medicine, The Catholic University of Korea, Seoul, Republic of Korea; ^3^Acryl, Seoul, Republic of Korea; ^4^Department of Medical Informatics, College of Medicine, The Catholic University of Korea, Seoul, Republic of Korea

**Keywords:** machine learning, depression, emotions, psychological interview, sentiment analysis

## Abstract

**Background:**

A psychiatric interview is one of the important procedures in diagnosing psychiatric disorders. Through this interview, psychiatrists listen to the patient’s medical history and major complaints, check their emotional state, and obtain clues for clinical diagnosis. Although there have been attempts to diagnose a specific mental disorder from a short doctor-patient conversation, there has been no attempt to classify the patient’s emotional state based on the text scripts from a formal interview of more than 30 min and use it to diagnose depression. This study aimed to utilize the existing machine learning algorithm in diagnosing depression using the transcripts of one-on-one interviews between psychiatrists and depressed patients.

**Methods:**

Seventy-seven clinical patients [with depression (*n* = 60); without depression (*n* = 17)] with a prior psychiatric diagnosis history participated in this study. The study was conducted with 24 male and 53 female subjects with the mean age of 33.8 (± 3.0). Psychiatrists conducted a conversational interview with each patient that lasted at least 30 min. All interviews with the subjects between August 2021 and November 2022 were recorded and transcribed into text scripts, and a text emotion recognition module was used to indicate the subject’s representative emotions of each sentence. A machine learning algorithm discriminates patients with depression and those without depression based on text scripts.

**Results:**

A machine learning model classified text scripts from depressive patients with non-depressive ones with an acceptable accuracy rate (AUC of 0.85). The distribution of emotions (surprise, fear, anger, love, sadness, disgust, neutral, and happiness) was significantly different between patients with depression and those without depression (*p* < 0.001), and the most contributing emotion in classifying the two groups was disgust (*p* < 0.001).

**Conclusion:**

This is a qualitative and retrospective study to develop a tool to detect depression against patients without depression based on the text scripts of psychiatric interview, suggesting a novel and practical approach to understand the emotional characteristics of depression patients and to use them to detect the diagnosis of depression based on machine learning methods. This model could assist psychiatrists in clinical settings who conduct routine conversations with patients using text transcripts of the interviews.

## Introduction

Depression is the most prevalent mental health issue that affects hundreds of millions of people and is considered one of the leading causes of burden globally (1, 2). It is estimated that the lifetime prevalence of depression among adults was 10.8% from 1994 to 2014 (3), and the burden due to mental disorders has not been reduced despite evidence-based interventions (1). In addition, the prevalence of depression in South Korea shows an increasing trend (4).

The diagnosis and evaluation of major depressive disorder (MDD) are based on diagnostic criteria based on DSM-5, which requires a clinical judgment of a trained clinician on listed symptoms, including depressed mood, markedly diminished interest or pleasure, significant weight loss, slowing down of thought, a reduction of physical movement, fatigue or loss of energy, reduced ability to think or concentrate, and recurrent thoughts of death (5). The screening for these symptoms mainly depends on diagnostic questionnaires such as Patient Health Questionnaire-9 (PHQ-9), Beck Depression Inventory (BDI) (6), and the Hamilton Depression Rating Scale (HDRS) (7). This questionnaire-based diagnostic approach necessitates an interview with clinicians, but it can be prone to biases as they are either self-reported by patients or administered by clinicians (8).

It is important to start treatment earlier for patients with MDD because the time to treatment is correlated with the prognosis (9). A diverse range of barriers, such as education, income, and accessibility, contribute to the underdiagnosis of depression (10). As an early diagnosis of depression may reduce the severe depressive symptoms and improve the prognosis, there is a need for an objective method that can diagnose patients’ emotional and depressive states.

Recent AI-based approaches have gained attraction to provide additional information on diagnosing depression. Physiological signals such as electroencephalogram (11, 12) and features from eye-blinking (13) were captured upon audio-visual stimuli to classify emotions by utilizing deep neural networks. More common approaches include applying deep learning models on audio and visual data from clinical patients and public datasets (14, 15), where widely used datasets classified facial expressions into emotional labels such as anger, disgust, fear, happiness, sadness, surprise, and neutral (16). Symptom severity of depression was measured based on the speech and 3D facial scan data in DAIC-WOZ dataset, and the convolutional neural network (CNN) model was reported to demonstrate reliable results in detecting MDD (14). Potential depression risk was tried to be identified on the video recordings of depression patients in China conducting structured tasks with a deep belief network (DBN) based model (15). There was also an audio-focused approach where patients’ low-level and high-level audio features were used to estimate depression severity scores and detect depression (17).

A series of studies have focused mainly on the acoustic and text features from the conversations (18, 19). Acoustic features in spontaneous speech were used to recognize depression against the normal control, with improvement was reported in the performance using the first few sentences (18). Indirect text features from the patients, such as the total number of sentences, average words spoken in each sentence, frequency of laughter, and depression-related words, were fed into the model in addition to audio and visual features (19). However, the nature of audio and video data requires much preparation for consistent recording quality across the samples (20), and even the laboratory setup to collect audiovisual data still requires extensive pre-processing to guarantee the quality of input into the model (21).

In addition, there have not been many attempts to measure symptom severity or identify depression by directly collecting data from the psychiatric interviews between the psychiatrists and the patients, where structured psychiatric interviews are essential in making an accurate diagnosis to satisfy the categorical conditions listed in DSM-5. The interviews are still often encouraged to induce free-of-context, unstructured conversations that can illicit subjective experiences from the patients (22), as such interviews are often the single most important source of information in obtaining clinical cues for psychiatrists.

In this study, we utilized XGBoost algorithm to identify depression based on the actual psychiatric interviews between the psychiatrists and the patients. We aimed to identify patients with depression against the psychiatric patients without depression based on the text scripts of routine psychotherapy sessions to overcome burdensome requirements in collecting and pre-processing the audiovisual data that have been widely used to analyze the depression patients with machine learning methods. We classified emotional characteristics of the text scripts from the interviews on the back of the improved accuracy of text emotion recognition applications (23–25). Transcripts from psychiatric interviews are easy to collect and require minimal pre-processing, whereas audio and visual data are more complex in nature and data processing perspective. It is one of the first attempts to identify depression using text emotion recognition based on routine psychiatric interviews in the clinical setting.

The rest of this paper is organized as follows: the data acquisition process from the clinical patients and the machine learning model were presented in Materials and Methods; results of depression classification is presented in Results; summary, future works, and limitations are discussed in Discussion; and lastly Conclusions.

## Materials and methods

### Participants

Seventy-seven clinical patients (24 male, 53 female) between 20 and 65 years old participated in this qualitative and retrospective study to develop a tool to detect depression. The dialogue data were acquired in a consecutive manner from all inpatients and outpatients who agreed to record their interview during the treatment. Participants were diagnosed with depression or anxiety, with or without a current episode, established through DSM-5. The clinical diagnosis was provided by the agreement of two or more psychiatrists at Seoul St. Mary’s Hospital by assessing the patients in person. Interviews with the participants were conducted from August 2021 to November 2022. All participants were required to provide informed consent forms to be considered as the subjects, and the Institutional Review Board of Seoul St. Mary’s Hospital approved this study (KC21ONSI0387).

Inclusion criteria included (1) adults aged 18–65 years; (2) individuals who have received a primary diagnosis of depression (ICD codes: F32, F33, F34) from the Department of Psychiatry and have undergone treatment; (3) for the control group, individuals who have not received the diagnoses or treatment mentioned in (2); and (4) individuals who have received sufficient explanation of this clinical trial, have understood it, voluntarily decided to participate, and provided written consent to adhere to precautions.

Exclusion criteria included any current or lifetime axis I psychiatric disorders, such as schizophrenia, schizoaffective disorder, other psychotic and substance-related disorders, organic mental disorders, neurological disorders (e.g., epilepsy, dementia), and cardiovascular disorders. A total of 10 people were excluded due to intake of prohibited substances such as alcohol and psychostimulant (*n* = 3), change in diagnosis (*n* = 5), and voluntary withdrawal of consent (*n* = 2).

### Patient characteristics

Among the 77 participants, 60 subjects were diagnosed with depression, and 17 subjects had other psychiatric illnesses (Table 1). The with-depression group included 16 males (26.7%) and 44 females (73.3%), whereas the without-depression group consisted of 8 males (47.1%) and nine females (52.9%). The mean age was 33.2 (±3.3) for the with-depression group and 35.9 (±6.9) for the control group. There were no significant differences in gender and age between the two groups (*p* > 0.05, Table 1).

**Table 1:** Patient characteristics.

		No. (%) of patients			
		Total (*n* = 77)	With- depression (*n* = 60)	Without- depression (*n* = 17)	*p*-value
Sex^a^					0.192
	Male	24 (31.2)	16 (26.7)	8 (47.1)	
	Female	53 (68.8)	44 (73.3)	9 (52.9)	
Age^b^					0.947
	Mean	33.8	33.2	35.9	
	(95% CI)	(30.8–36.8)	(29.9–36.5)	(29.0–42.7)	
	20–29	42 (54.5)	33 (55.0)	9 (52.9)	
	30–39	16 (20.8)	13 (21.7)	3 (17.6)	
	40–49	5 (6.5)	4 (6.7)	1 (5.9)	
	50–59	9 (11.7)	6 (10.0)	3 (17.6)	
	60+	5 (6.5)	4 (6.7)	1 (5.9)	
	Minimum	20	20	20	
	Maximum	64	64	63	
Diagnosis					
	Adjustment disorder with depressed mood	3 (3.9)	3 (5.0)		
	Bipolar disorder (currently depression)	11 (14.3)	11 (18.3)		
	Major depressive disorder	22 (28.6)	22 (36.7)		
	Persistent depressive disorder	22 (28.6)	22 (36.7)		
	Other specified depressive disorder	2 (2.6)	2 (3.3)		
	Anxiety disorder	1 (1.3)		1 (5.9)	
	Anorexia nervosa	1 (1.3)		1 (5.9)	
	Acute stress disorder	1 (1.3)		1 (5.9)	
	Alochol use disorder	3 (3.9)		3 (17.6)	
	Bipolar and related disorder	1 (1.3)		1 (5.9)	
	Intermittent explosive disorder	1 (1.3)		1 (5.9)	
	Post-traumatic stress disorder	6 (7.8)		6 (35.3)	
	Somatic symptom disorder	1 (1.3)		1 (5.9)	
	Substance use disorder	1 (1.3)		1 (5.9)	
	Trichotillomania	1 (1.3)		1 (5.9)	

^a^ Chi-squared test on with-depression group vs. without-depression group.

^b^ Fisher’s exact test on with-depression group vs. without-depression group.

### Data acquisition

A psychiatrist performed a psychiatric interview with each subject in a quiet psychiatric consultation room. The interviews were conducted as part of psychotherapy, in the form of semi-structured format which included typical attributes such as daily lives, chief complaints, thought contents, cognitions, judgments, and insights. The interviews lasted 30 min or longer. All interviews were recorded under the subjects’ consent, and text scripts were produced by a separate scripter for the first 15 to 20 min of the voice recordings after each interview.

Then, sentences from psychiatrist were removed from the text scripts so that only the sentences from the subjects could be left in the scripts. Emotional classification of each sentence was conducted by Emotional Analysis Module patented by Acryl Inc. at the Republic of Korea Intellectual Property Office (26), where the input is a single sentence, and the output is a list of probabilities of 8 emotions of the corresponding sentence, namely surprise, fear, anger, love, disgust, sadness, neutral, and happiness. For each transcript, probabilities of eight emotions were derived for the first 250 sentences, resulting in 2,000 probability data. The average probability value for each emotion was calculated and appended as statistics in front of the 2,000 data. As a result, 2,008 probability data were formed as vectors and became the input vector for the machine learning model.

The transcription and feeding of the input vectors into the machine learning model was conducted until the model to detect depression was believed to perform with adequate accuracy.

### Machine learning model to detect depression

Boosting is an ensemble method to create a strong learner by combining multiple weak learners. A weak learner indicates a model that performs slightly better than a randomized prediction. In contrast, a strong learner suggests a model that performs well, significantly better than a randomized prediction. A model is iteratively modified to minimize a loss function by evaluating errors from the previous model and adjust the weights to “boost” the accuracy, but overfitting can remain as a problem (27).

XGBoost is an algorithm that combines multiple decision trees to make predictions (28) based on Gradient Boosting Model (GBM) to overcome the overfitting problem by adopting Classification and Regression Tree (CART) model for regression. It also makes predictions extremely fast by parallel processi3ng of the data. In addition, a weighted quantile sketch was used to handle missing data.

The 166 scripts were split in training and test sets using scikit-learn package, which uses the stratified random sampling method, into an 80/20 ratio. 4-fold cross-validation was conducted on the training set to prevent overfitting (29). Hyperparameters, including learning rate, maximum depth, regularization factor (lambda), early stopping, and evaluation metric, were optimized using grid search (30).

The performance of a model was evaluated with Accuracy and F1 score. Accuracy is the percentage of correct predictions made, but it can sometimes be misleading when the dataset is unbalanced. The F1 score is a harmonic mean of precision and recall, reflecting the imbalance of the dataset. In addition, Area Under the Curve (AUC) was also evaluated, where in general, AUC under 0.7 indicates less reliable, AUC between 0.7 and 0.8 shows somewhat reliable, and more than 0.8 means highly reliable.

RStudio 2022.12.0 + 353 was used for the statistical analysis of the data collected.

## Results

### Characteristics of extracted sentences

A total of 451 scripts were originally collected from the 77 subjects. The scripts were pre-processed in the form appropriate for learning the model. To avoid overweighting a particular diagnosis or subject, the emotion vectors collected from the first five scripts from each subject were selected in the sequential order and used for analysis to avoid oversampling, as the average number of scripts collected from the subjects was 5.8. As a result, 166 scripts were eventually fed into the model to detect depression.

As a result, a total of 20,405 sentences were split from the 166 scripts, and an emotion with the highest probability was considered as the representative emotion of each sentence in comparing emotional characteristics of the two groups. In the with-depression group, there were 15,223 sentences with an average of 2,184 words consisting of 8,072 characters on each script. There were 5,182 sentences with an average of 2,171 words and 8,156 characters on each script in the without-depression group.

### Distribution of emotions

The frequently represented emotions in the with-depression group were neutral (59.8%), sadness (16.3%), disgust (10.7%), fear (7.3%), and happiness (4.1%). The without-depression group had a similar order of the frequently represented emotions, namely neutral (57.1%), sadness (15.8%), disgust (14.1%), fear (7.9%), and happiness (3.5%). The distribution of eight emotions represented by the sentences significantly differed between the two groups based on the Chi-squared test of homogeneity (*p* < 0.001, Table 2).

**Table 2:** Emotions counts from the scripts.

		No. (%) of sentences			
		Total	With- depression	Without- depression	*p*-value^a^
Emotions classified					<0.001
	Surprise	146 (0.7)	113 (0.7)	33 (0.6)	
	Fear	1,519 (7.4)	1,112 (7.3)	407 (7.9)	
	Anger	153 (0.7)	107 (0.7)	46 (0.9)	
	Love	45 (0.2)	39 (0.3)	6 (0.1)	
	Sadness	3,304 (16.2)	2,487 (16.3)	817 (15.8)	
	Disgust***	2,357 (11.6)	1,626 (10.7)	731 (14.1)	
	Neutral**	12,069 (59.1)	9,110 (59.8)	2,959 (57.1)	
	Happiness	812 (4.0)	629 (4.1)	183 (3.5)	
Total		20,405 (100.0)	15,223 (100.0)	5,182 (100.0)	

^a^ Chi-squared test on with-depression group vs. without-depression group.

**p < 0.01 based on post-hoc analysis of Pearson’s Chi-squared test.

***p < 0.001 based on post-hoc analysis of Pearson’s Chi-squared test.

Disgust (*p* < 0.001) and neutral (*p* < 0.01) were identified as the emotions that contributed to the significant difference in the distributions between the two groups based on the *post hoc* analysis of the residuals of the chi-squared test (31).

### Classification results of with-depression and without-depression groups

**Figure 1:**
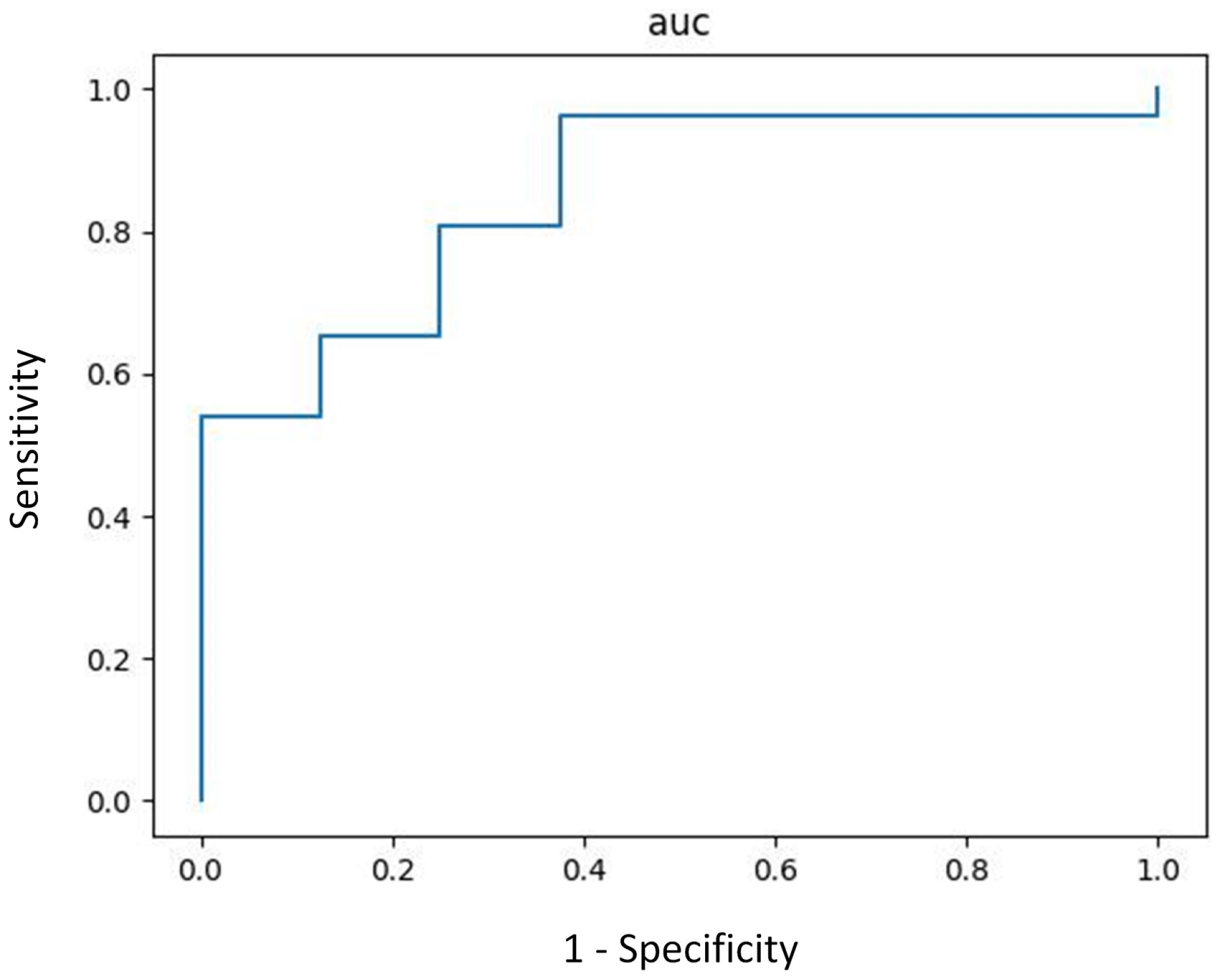
ROC curve on the test set.

The ROC curve of the machine learning model which used the original probability vectors showed an AUC of 0.85 (Figure 1) upon the hyperparameters optimized with grid search (32). The model classified patients with depression against those without depression with a sensitivity of 0.96, specificity of 0.25, an accuracy of 0.79, and an F1 score of 0.88 (Table 3).

**Table 3:** Confusion matrix on the test set.

		Ground truth	
		With-depression	Without-depression
Model output	With-depression	73.5% (25)	17.6% (6)
	Without-depression	2.9% (1)	5.9% (2)

* Key metrics of classification include accuracy of 79.4%, F1 score of 87.7%, sensitivity of 96.2%, and specificity of 25.0%. F1 score is defined by 2 * Precision * Recall / (Precision + Recall).

## Discussion

Our text emotion recognition algorithm revealed the difference in emotion distributions between the patients with depression and the control group. The distribution of emotions extracted from the sentences showed significant differences between the two groups, mainly due to less frequent expressions of disgust in the with-depression group. The machine learning model could classify patients with depression against the without-depression control with good reliability based on the emotional profiles extracted from the transcripts.

Among eight emotional labels (surprise, fear, anger, love, disgust, sadness, neutral, and happiness), the most contributing emotion that discriminates between depression and the control was disgust. Patients with depression were known to have problems recognizing facial expressions showing disgust (33, 34). Functional MRI signals responded in higher intensity among patients with depression to disgust (33), suggesting impaired functioning in the basal ganglia (34). Depression is associated with self-disgust (35), presumably due to altered emotion regulation strategies (36). In addition to the recognition of and response to external stimuli, the findings of this study concur with the association of expression of disgust and depression. Neutral was one of the contributing factors in discriminating the two groups.

Depression is typically characterized by a depressed mood or sadness, but its contribution in discriminating the two groups in this study was not significant. The mood or the sadness is generally determined by physicians from the general atmosphere throughout the conversation. Meanwhile in this study, “sadness” as an emotion was derived from specific sentences, indicating sentiments at certain moments during the conversation. Sadness was not significant in our study due to the difference of the time-interval between the clinical cue and our method. In addition, we compared the with-depression group against the patients without depression, not against the normal control group. Some patients in the control group such as those with PTSD and somatic symptoms disorder, might have expressed sadness as much as depression group under the influence of accompanying symptoms.

The probability vectors of emotions derived from sentences were fed to train the machine learning model, and the model discriminated depression from the control group with an AUC of 0.85, indicating a high reliability of the model. Feature importance analysis revealed that the model did not depend solely on any single emotion in detecting the depression, and the probability vectors of the sentences from the early part of the interviews were considered more important by the model compared to the latter part of the interviews (Table 4). Feature importance represents the contribution of each input feature in making branches in the decision tree. It is evaluated by the change in the model performance given the exclusion of a certain input feature.

**Table 4:** Feature importance analysis.

		Importance
By emotions	Neutral	0.187
	Love	0.155
	Fear	0.153
	Surprise	0.134
	Anger	0.120
	Disgust	0.094
	Happiness	0.079
	Sadness	0.077
By location of sentences (n^th^ sentence)	1–20	0.317
	81–100	0.186
	21–40	0.151
	61–80	0.110
	41–60	0.103
	101–120	0.054
	141–160	0.043
	121–140	0.036
	161 and later	0.000

Previous studies have normally used audio and visual dataset as inputs to detect depression and its severity (14, 15, 17, 18), but the nature of audiovisual data poses hurdles in contemplating clinical applications for psychiatrists (20, 21). In contrast, text data in the form of transcripts of conversations based on the recordings of routine psychiatric interviews, as collected in this study, is incomparably easier to obtain upon the subject’s consent. An ordinary voice recorder in the office and a mean to transcribe of the conversation would suffice the setting for the data collection and the audio-to-text pre-processing. Such a simple requirement to generate the model input suggests a great advantage in applying to clinical situations.

Considering the objective of this study to assist psychiatrists in the actual clinical situations, the model should be able to detect subtlety of depression that psychiatrists might have missed. Currently, the model provides relatively low specificity compared to its very high sensitivity. While we recognize the need to demonstrate improved overall performance of the model, we also believe that the advantage of high sensitivity outweighs any disadvantage posed by the low specificity, as early recognition and proper intervention are important in treating depression with better outcomes (37).

There are several limitations to this study. First, psychotherapy sessions are semi-structured and conducted by multiple psychiatrists of the hospital depending on the availability. This would have allowed flexibility to explore deeper into the thoughts and emotions brought up by the patients depending on the flow of the conversation. Such less standardized interviews were thus considered more suitable for this study. However, psychotherapy sessions are less standardized and more difficult to quantify, and the questions and contents may vary depending on the interviewers. Structured interviews could have improved the credibility of the probability vectors of the emotions derived from the interviews.

Also, the random split of input data by scikit learn package might have resulted in the scripts from the same person being put into both the training and test set, considering the dataset size for this study. The model could have been trained in a way that classifies depression based on the person’s traits rather than the traits of the depression itself. A larger dataset could improve the model, not only in terms of the overall performance, including sensitivity, but also by minimizing the possibility of learning any individual’s trait so that the model ultimately identifies the depression solely based on the emotional features of depression.

There are a couple of factors that might have affected the external validity of this study. The number of data is limited due to the retrospective nature of the study, and the model’s performance along with statistical power could have improved further by feeding model inputs. Also, the control group consisted of psychiatric patients without depression, rather than non-clinical samples without any psychiatric diagnosis. It would have been valuable if such non-clinical samples were also recruited to compare against the with-depression group. However, we believe that it is more difficult to detect patients with depression against the patients with other psychiatric diagnosis, as conducted in this study. In addition, the subjects in the with-depression group and the without-depression control group were not exactly matched due to the retrospective nature of this study. We plan to test the detection algorithm on non-clinical subjects in the future in a prospective manner.

The number of scripts collected for this study was originally much larger than that of the input scripts fed into the model. We decided to use a maximum of 5 scripts for each subject to avoid potential bias due to oversampling. For example, we collected more than 40 scripts from five subjects, three from the with-depression group and the rest from the without-depression control group. It could have improved the performance metrics of the model when the entire data collection was used, but the risk associated with depending on a few subjects should be avoided. Collecting an evenly distributed number of scripts from the subjects would improve the model’s performance and avoid bias arising from the oversampling.

Acryl’s Emotional Analysis Module, which was used to derive probability vectors assigned to the sentences of the text scripts, did not consider any context or meanings of the sentence. Large Language Models (LLM) has been increasingly used recently in many applications which can consider textual contexts based on the parameters and datasets much larger than the conventional models in analyzing text data. It remains as a future work to incorporate LLM in the process of classifying emotions from the text scripts.

## Conclusion

This study suggests a novel approach to detect depression with conversational scripts with patients based on text emotion recognition and a machine learning model. Emotional distribution significantly differed between the depression and the control group, and the model showed a reliable performance in classifying patients with depression from those without depression. Our results could assist clinicians in the initial diagnosis and follow-up of depressive patients with conventional diagnostic tools. Further studies would improve the performance, potentially detecting depression alongside the psychiatrists in the clinics and hospitals.

## Data availability statement

The raw data supporting the conclusions of this article will be made available by the authors, without undue reservation.

## Ethics statement

The studies involving humans were approved by the Institutional Review Board of Seoul St. Mary’s Hospital. The studies were conducted in accordance with the local legislation and institutional requirements. The participants provided their written informed consent to participate in this study.

## Author contributions

JO: Conceptualization, Data curation, Formal analysis, Methodology, Writing – original draft, Writing – review & editing, Supervision, Investigation. TL: Formal analysis, Validation, Writing – original draft, Writing – review & editing, Investigation, Methodology. EC: Writing – original draft, Investigation. HyoK: Formal analysis, Visualization, Writing – review & editing. KC: Formal analysis, Visualization, Writing – review & editing. HyuK: Formal analysis, Visualization, Writing – review & editing. JC: Data curation, Writing – review & editing. H-HS: Data curation, Writing – review & editing. JL: Writing – review & editing. IC: Writing – review & editing. D-JK: Conceptualization, Data curation, Funding acquisition, Supervision, Writing – review & editing.
